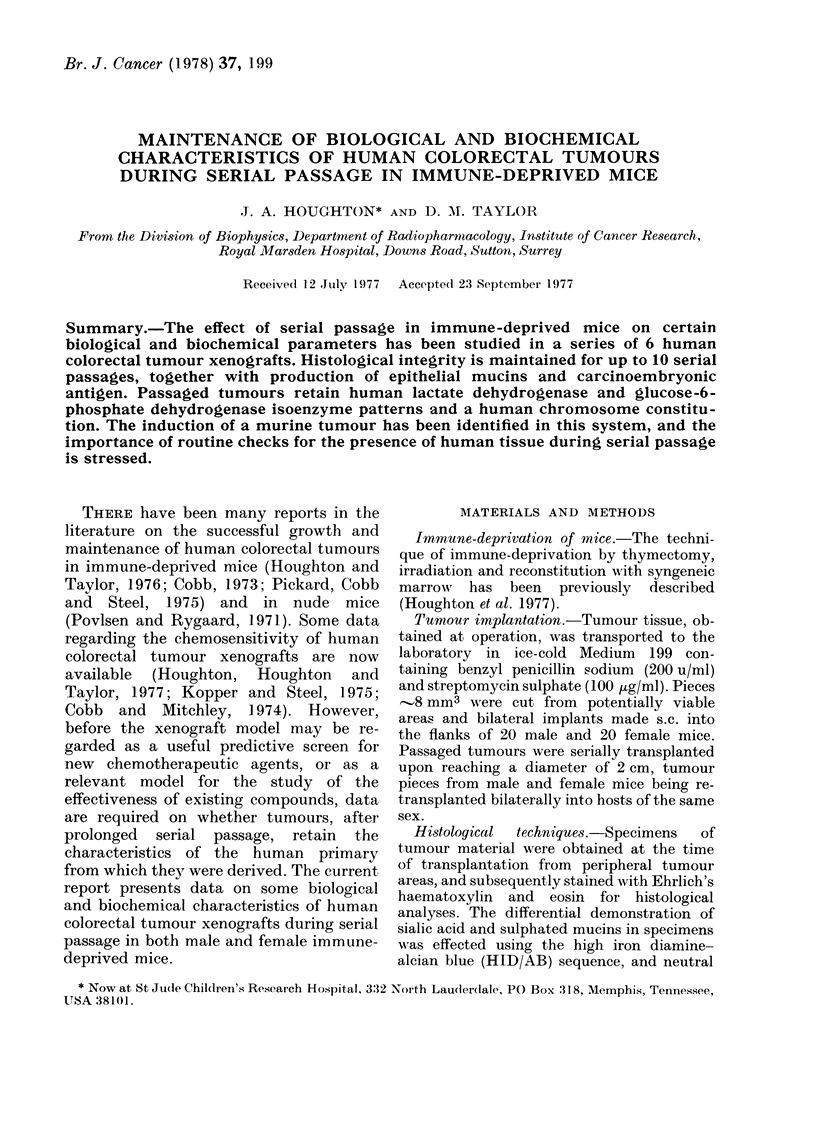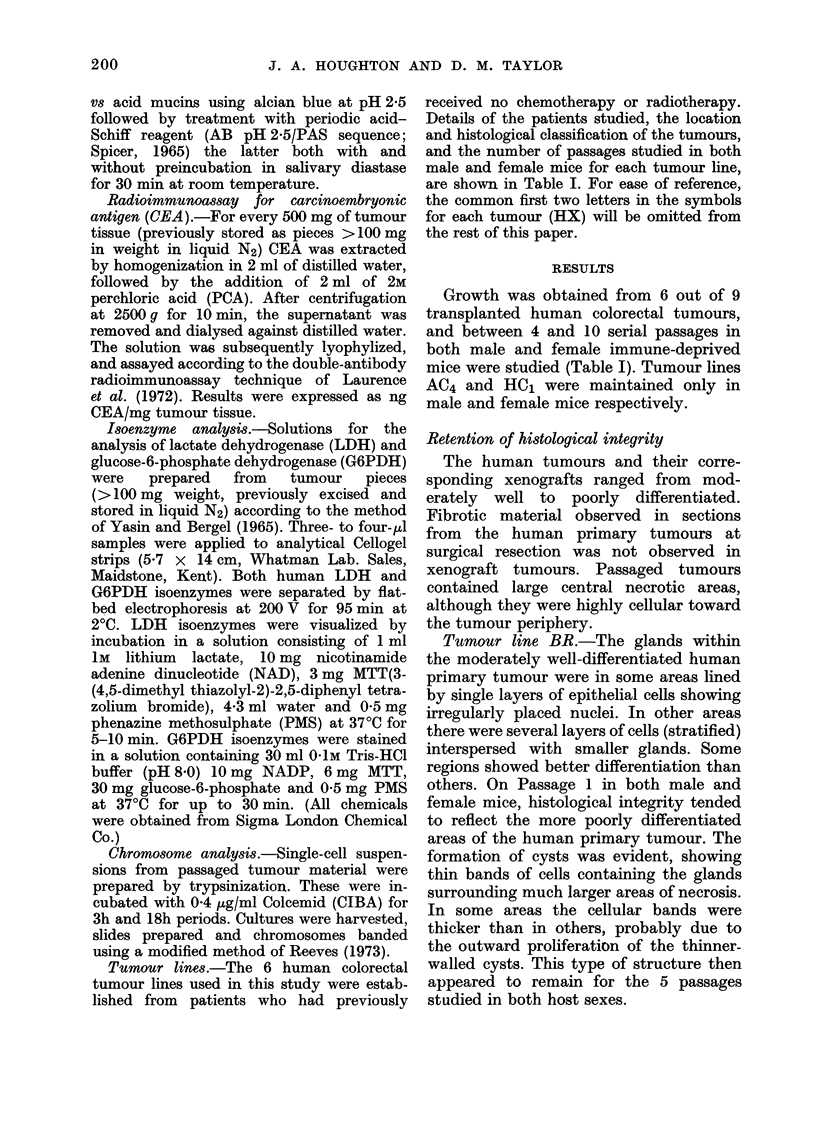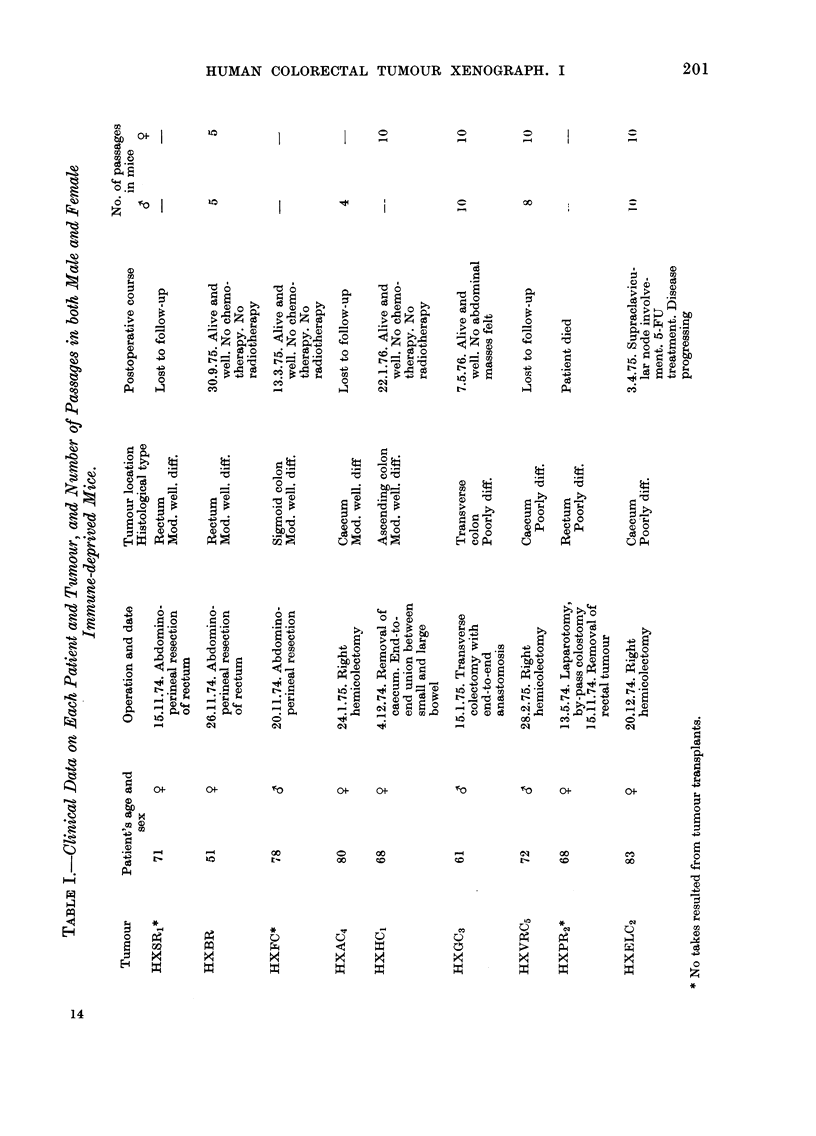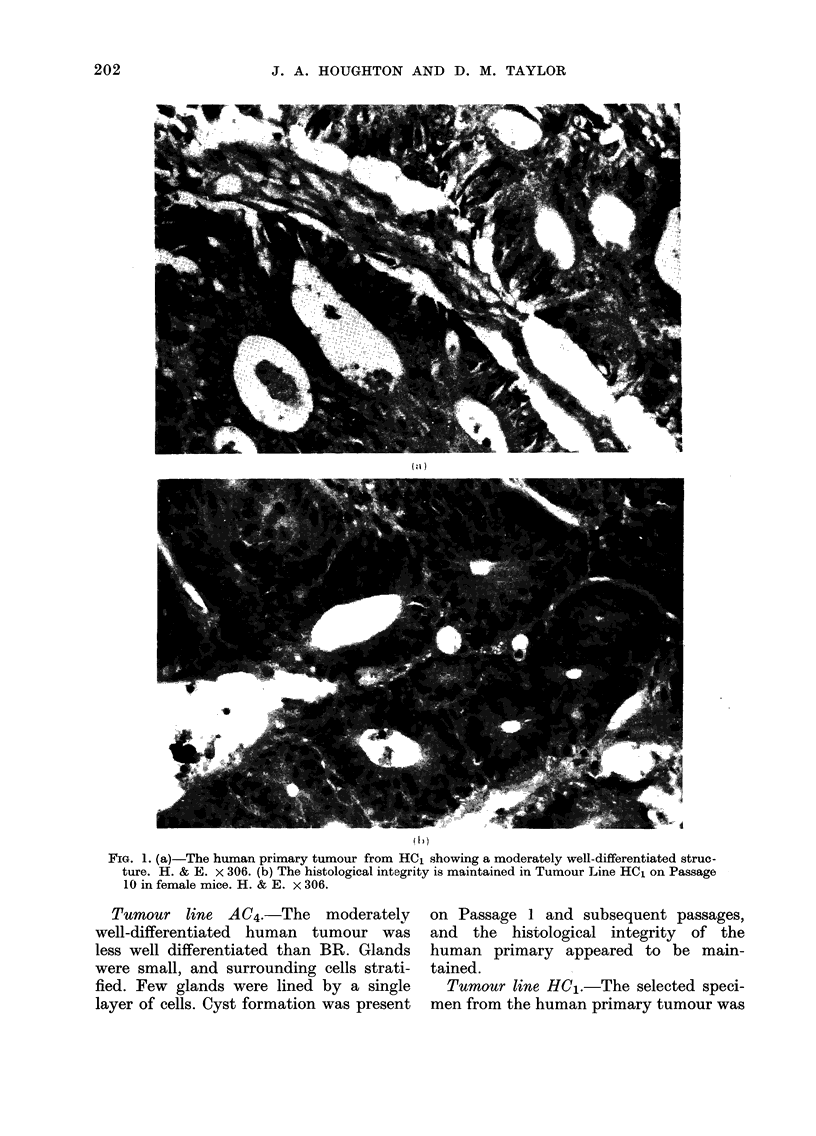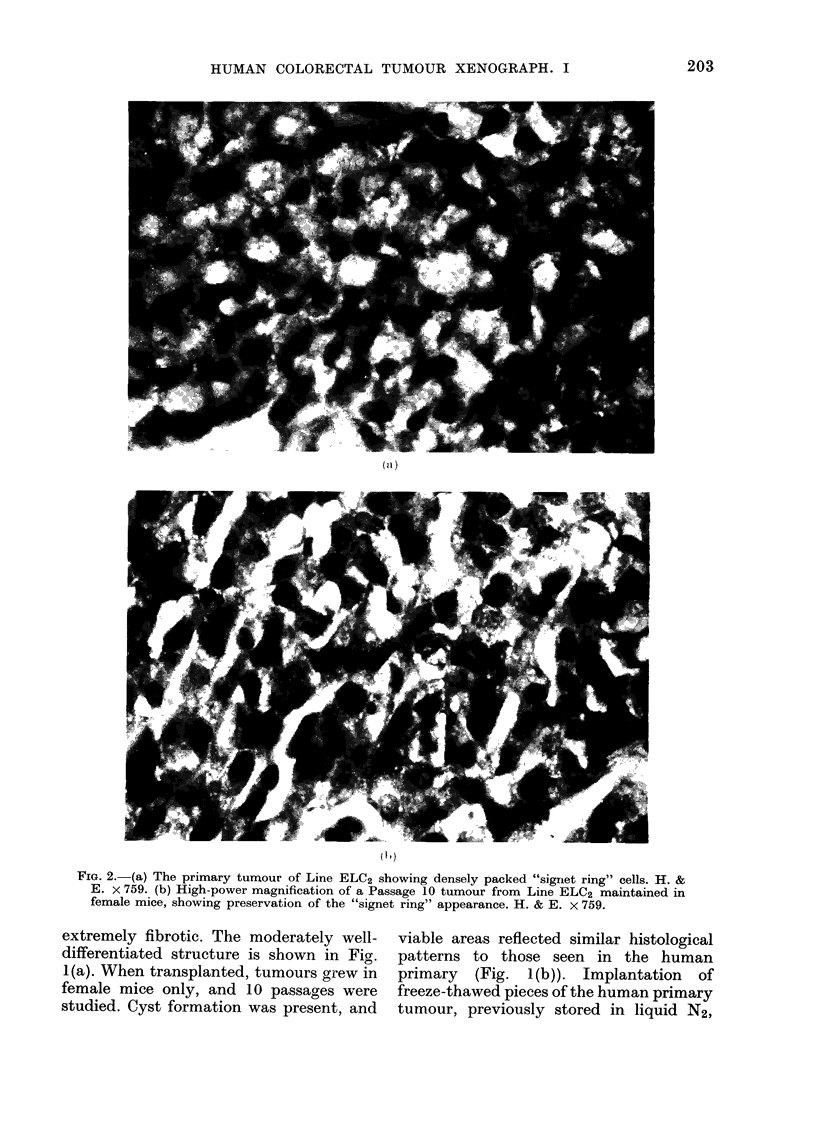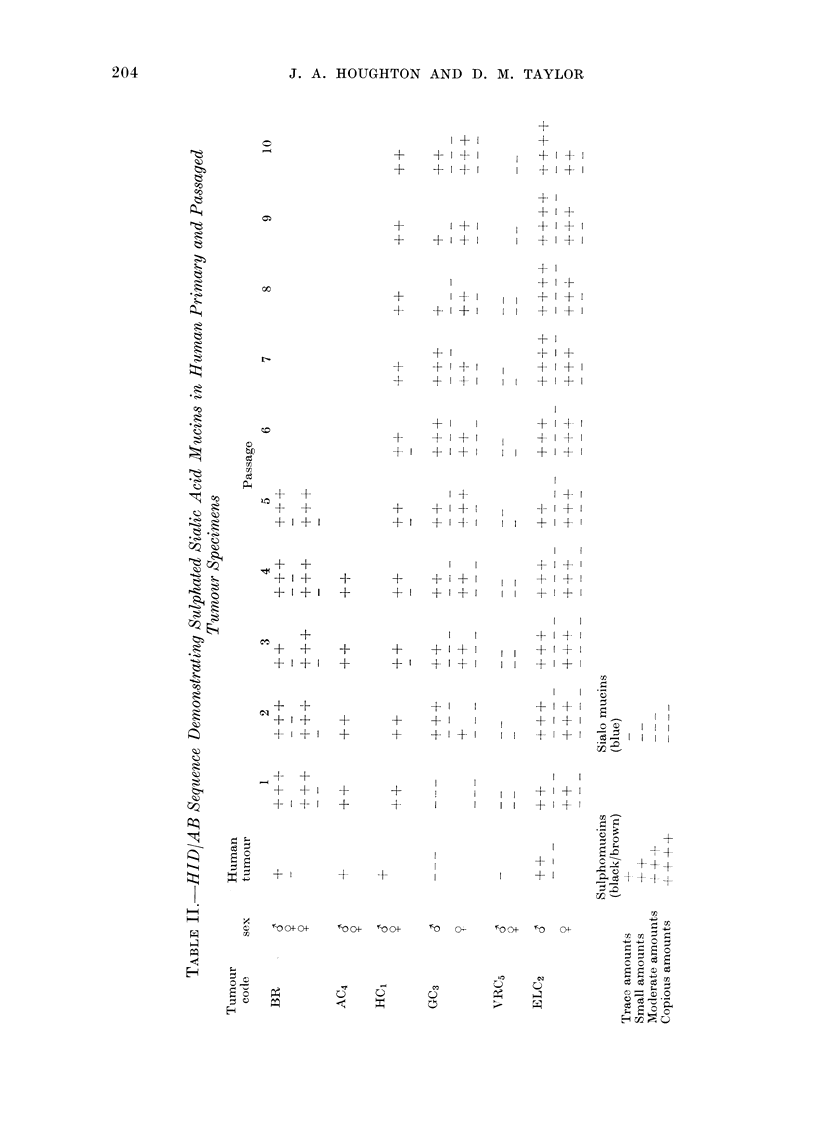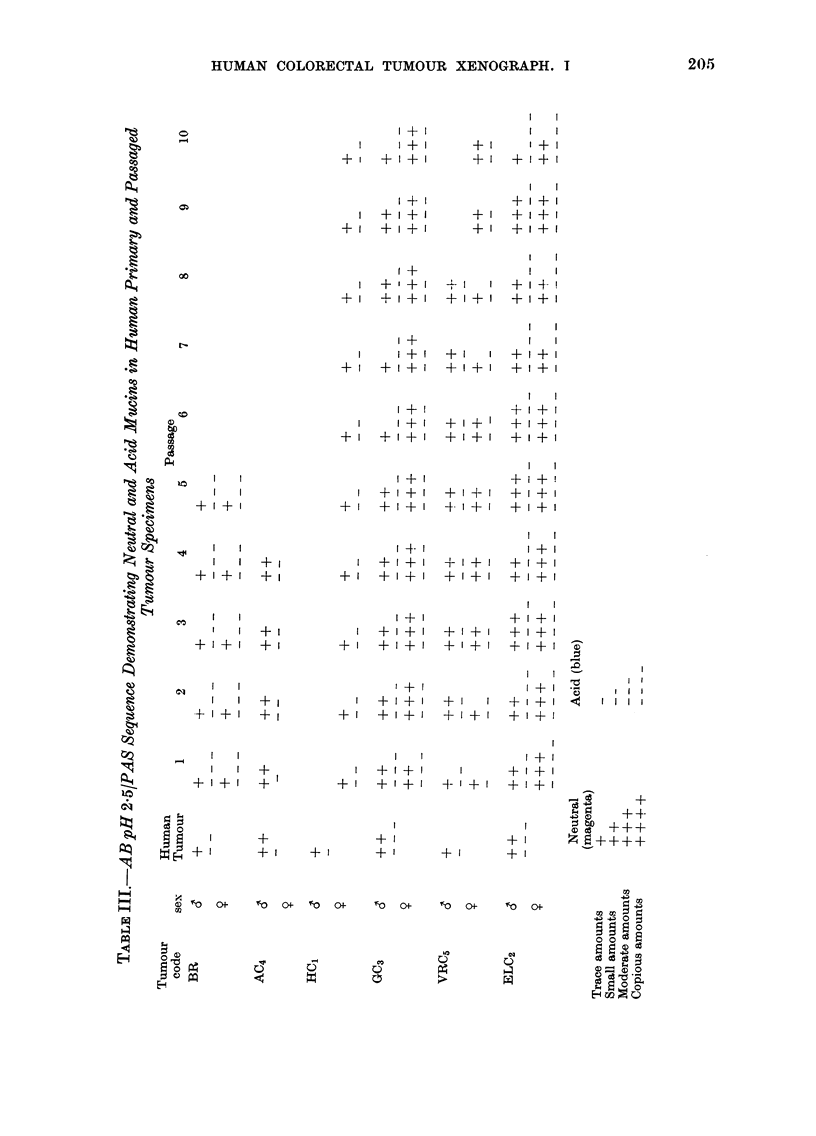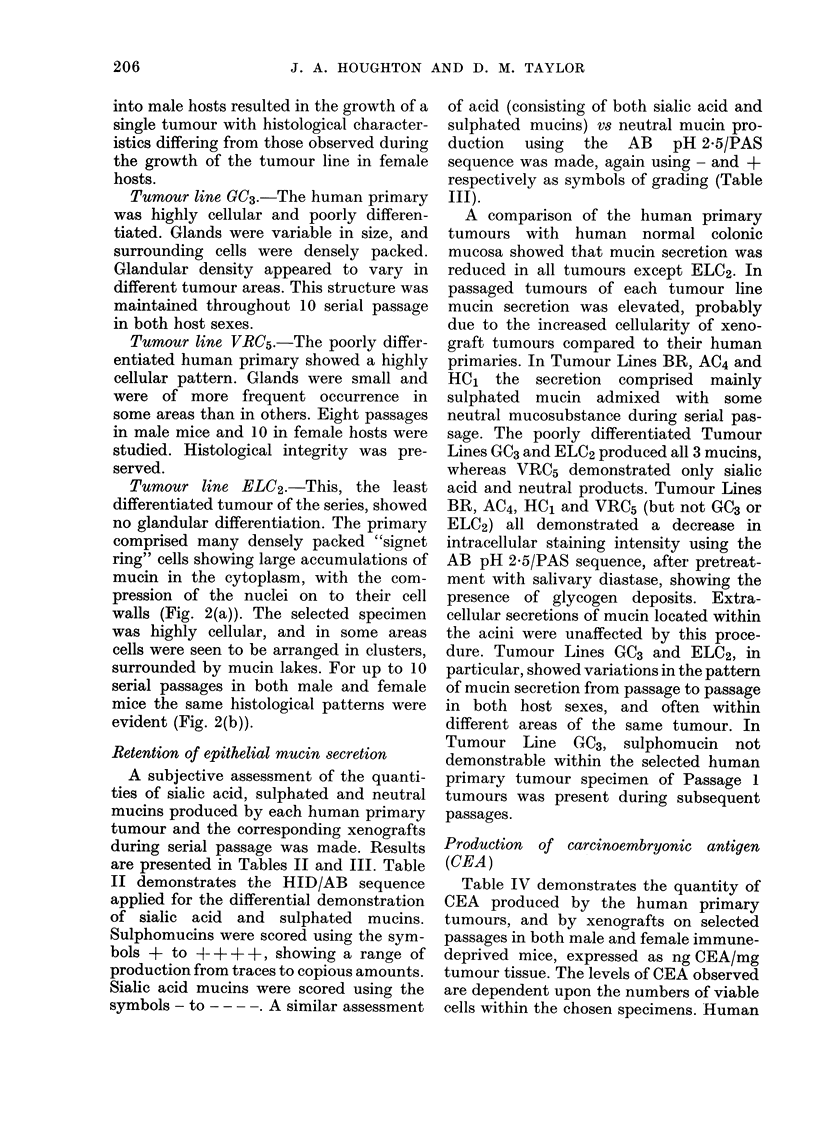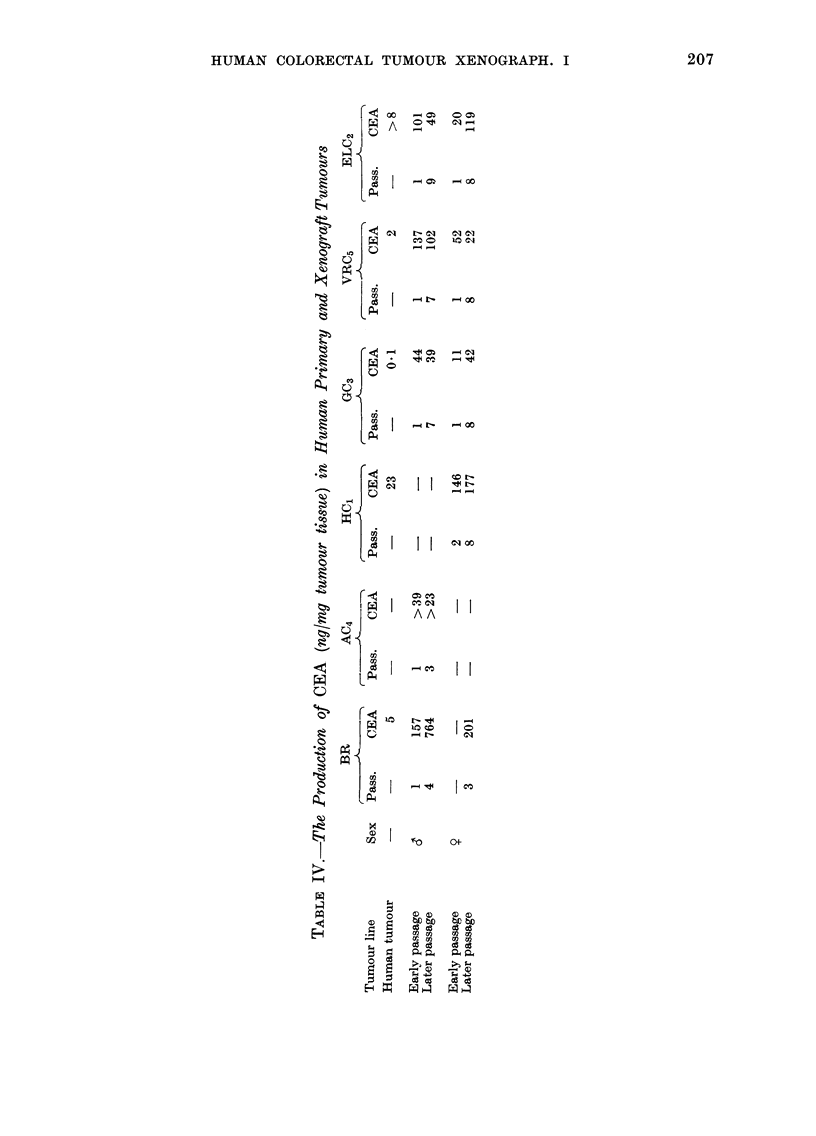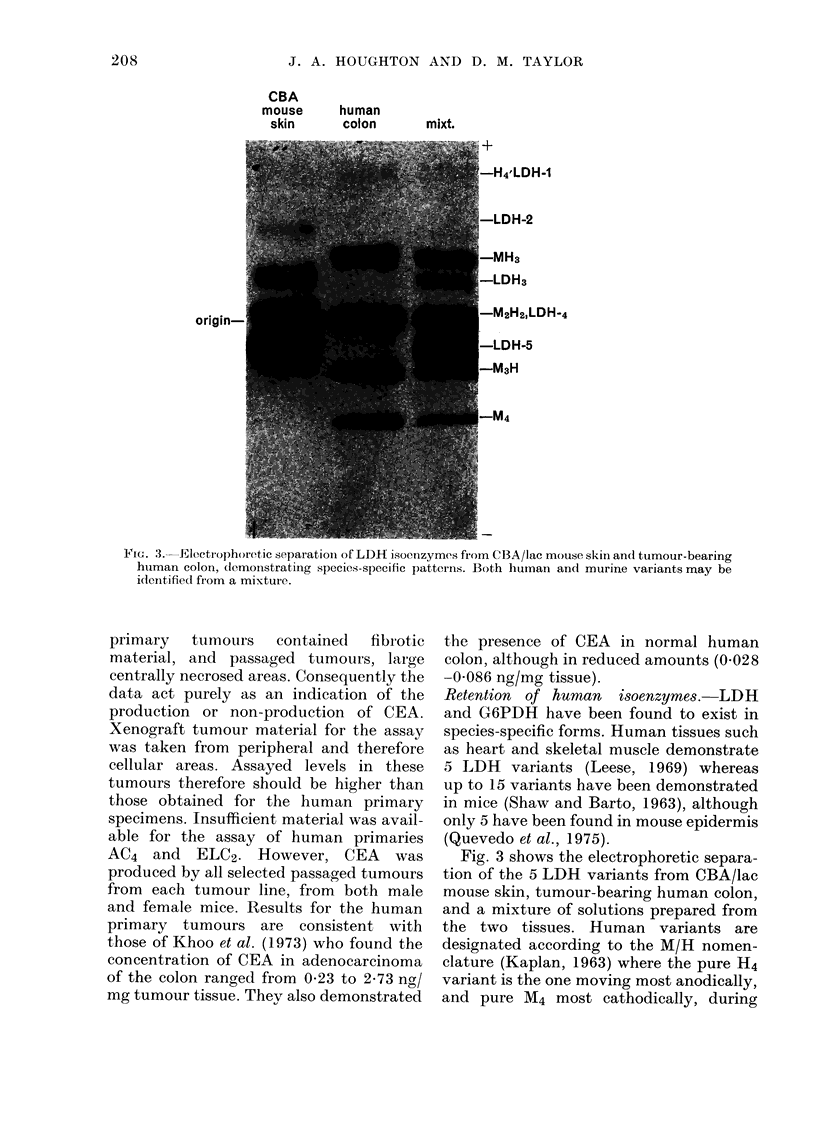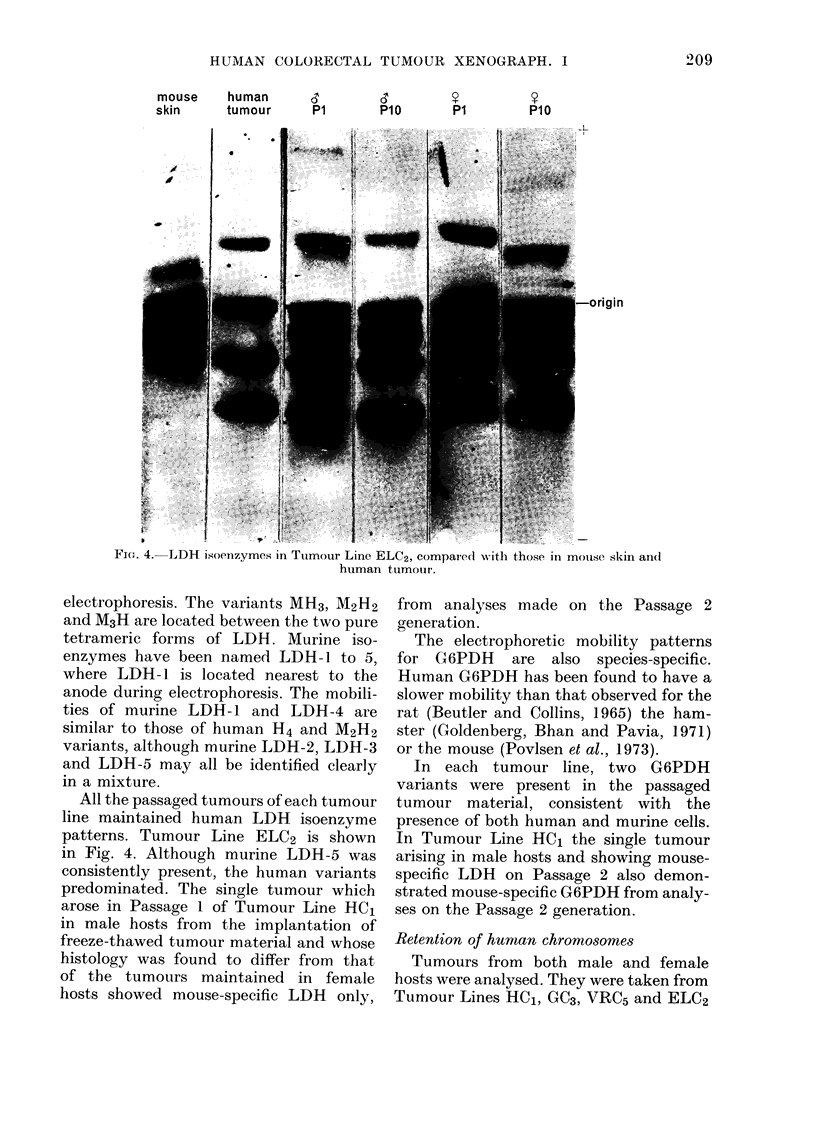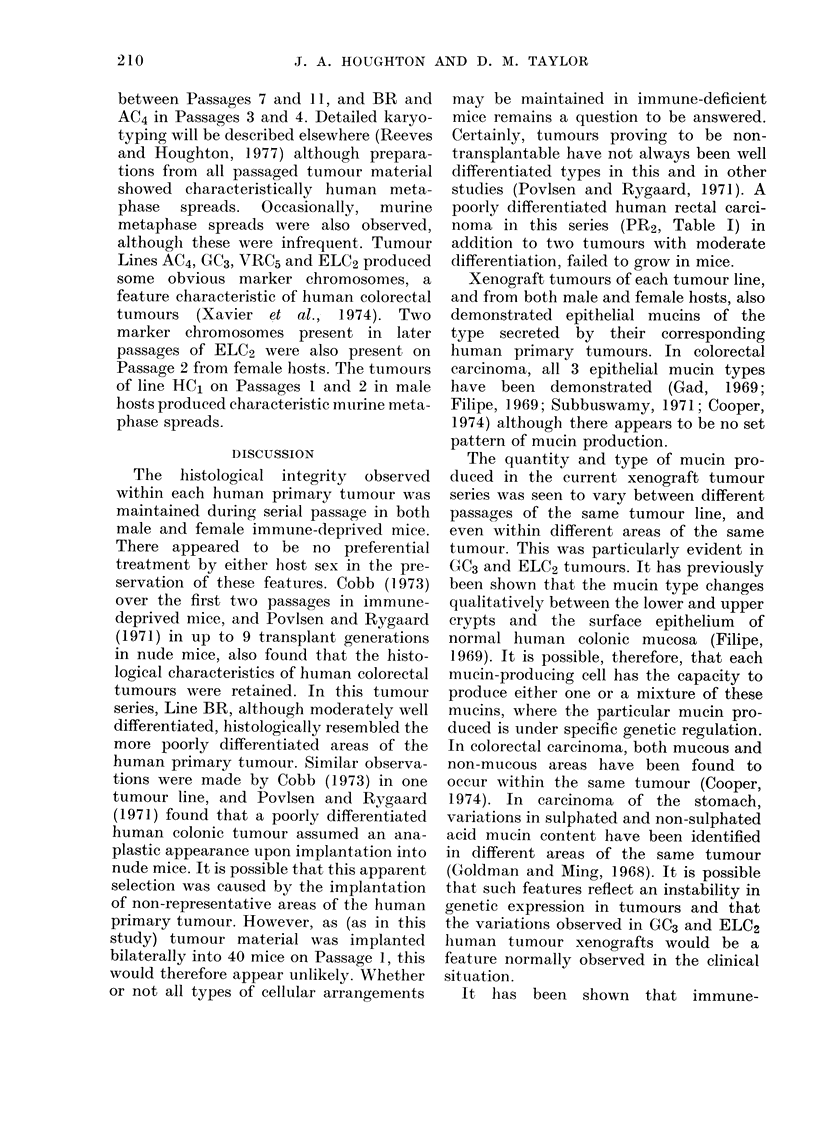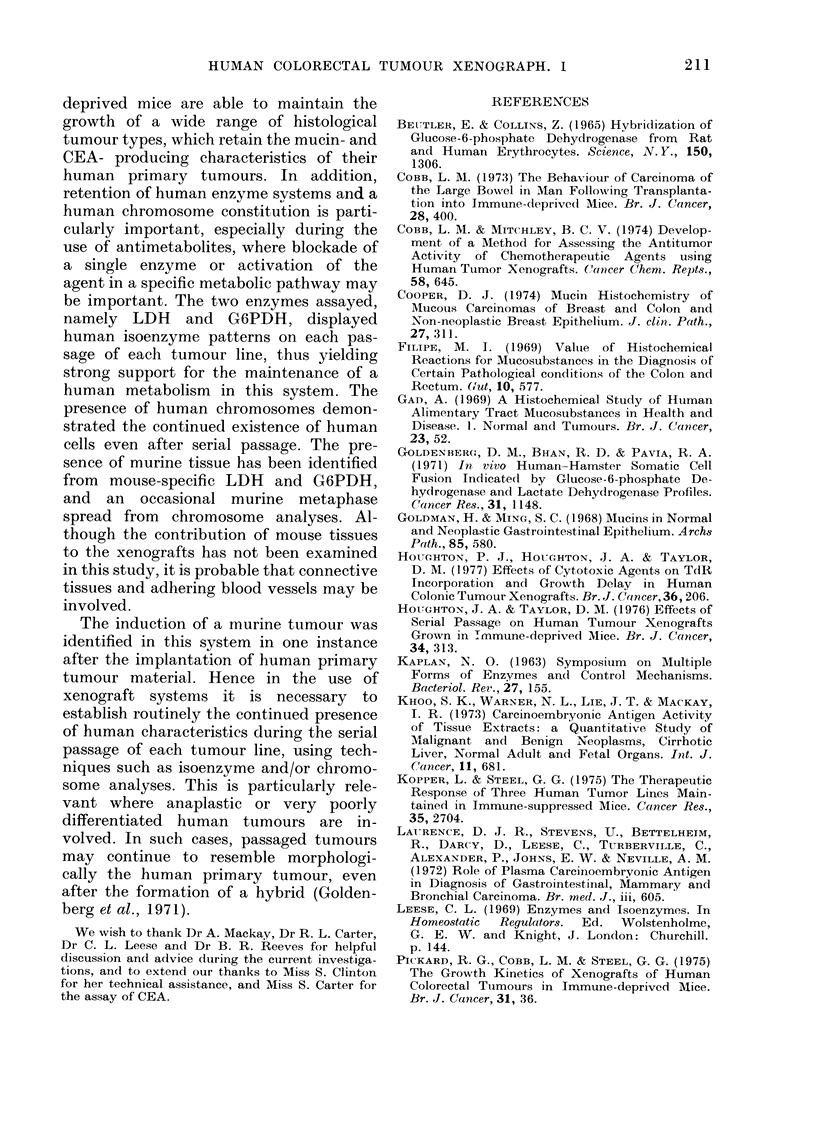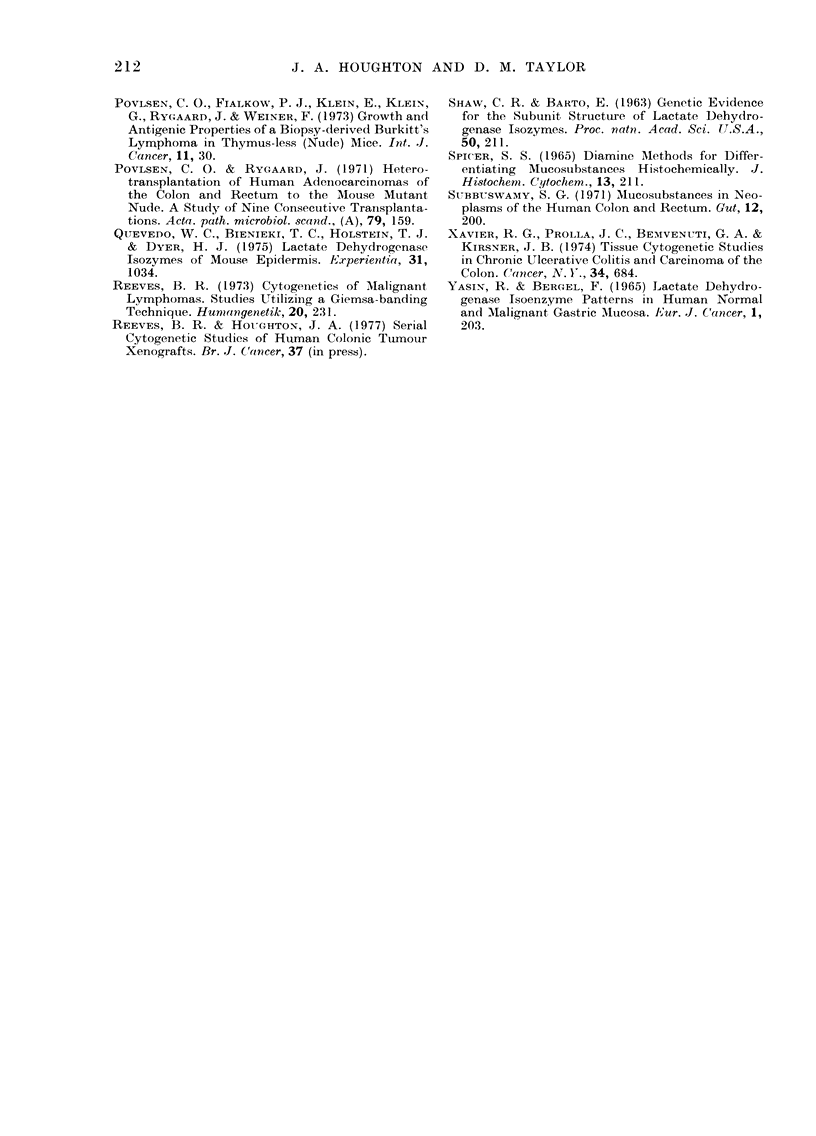# Maintenance of biological and biochemical characteristics of human colorectal tumours during serial passage in immune-deprived mice.

**DOI:** 10.1038/bjc.1978.28

**Published:** 1978-02

**Authors:** J. A. Houghton, D. M. Taylor

## Abstract

**Images:**


*Br. J. Cancer* (1978) **37**, 199

### MAINTENANCE OF BIOLOGICAL AND BIOCHEMICAL CHARACTERISTICS OF HUMAN COLORECTAL TUMOURS DURING SERIAL PASSAGE IN IMMUNE-DEPRIVED MICE

J. A. HOUGHTON* AND D. M. TAYLOR

*From the Division of Biophysics, Department of Radiopharmacology, Institute of Cancer Research, Royal Marsden Hospital, Downs Road, Sutton, Surrey*

Received 12 July 1977 Accepted 23 September 1977

**Summary.—The effect of serial passage in immune-deprived mice on certain biological and biochemical parameters has been studied in a series of 6 human colorectal tumour xenografts. Histological integrity is maintained for up to 10 serial passages, together with production of epithelial mucins and carcinoembryonic antigen. Passaged tumours retain human lactate dehydrogenase and glucose-6-phosphate dehydrogenase isoenzyme patterns and a human chromosome constitution. The induction of a murine tumour has been identified in this system, and the importance of routine checks for the presence of human tissue during serial passage is stressed.**

THERE have been many reports in the literature on the successful growth and maintenance of human colorectal tumours in immune-deprived mice (Houghton and Taylor, 1976; Cobb, 1973; Pickard, Cobb and Steel, 1975) and in nude mice (Povlsen and Rygaard, 1971). Some data regarding the chemosensitivity of human colorectal tumour xenografts are now available (Houghton, Houghton and Taylor, 1977; Kopper and Steel, 1975; Cobb and Mitchley, 1974). However, before the xenograft model may be regarded as a useful predictive screen for new chemotherapeutic agents, or as a relevant model for the study of the effectiveness of existing compounds, data are required on whether tumours, after prolonged serial passage, retain the characteristics of the human primary from which they were derived. The current report presents data on some biological and biochemical characteristics of human colorectal tumour xenografts during serial passage in both male and female immune-deprived mice.

### MATERIALS AND METHODS

*Immune-deprivation of mice.*—The technique of immune-deprivation by thymectomy, irradiation and reconstitution with syngeneic marrow has been previously described (Houghton *et al.* 1977).

*Tumour implantation.*—Tumour tissue, obtained at operation, was transported to the laboratory in ice-cold Medium 199 containing benzyl penicillin sodium (200 u/ml) and streptomycin sulphate (100 $\mu$g/ml). Pieces $\sim$8 mm$^3$ were cut from potentially viable areas and bilateral implants made s.c. into the flanks of 20 male and 20 female mice. Passaged tumours were serially transplanted upon reaching a diameter of 2 cm, tumour pieces from male and female mice being retransplanted bilaterally into hosts of the same sex.

*Histological techniques.*—Specimens of tumour material were obtained at the time of transplantation from peripheral tumour areas, and subsequently stained with Ehrlich's haematoxylin and eosin for histological analyses. The differential demonstration of sialic acid and sulphated mucins in specimens was effected using the high iron diamine–alcian blue (HID/AB) sequence, and neutral

* Now at St Jude Children's Research Hospital, 332 North Lauderdale, PO Box 318, Memphis, Tennessee, USA 38101.

*vs* acid mucins using alcian blue at pH 2·5 followed by treatment with periodic acid–Schiff reagent (AB pH 2·5/PAS sequence; Spicer, 1965) the latter both with and without preincubation in salivary diastase for 30 min at room temperature.

*Radioimmunoassay for carcinoembryonic antigen (CEA).*—For every 500 mg of tumour tissue (previously stored as pieces >100 mg in weight in liquid $N_2$) CEA was extracted by homogenization in 2 ml of distilled water, followed by the addition of 2 ml of 2M perchloric acid (PCA). After centrifugation at 2500 *g* for 10 min, the supernatant was removed and dialysed against distilled water. The solution was subsequently lyophylized, and assayed according to the double-antibody radioimmunoassay technique of Laurence *et al.* (1972). Results were expressed as ng CEA/mg tumour tissue.

*Isoenzyme analysis.*—Solutions for the analysis of lactate dehydrogenase (LDH) and glucose-6-phosphate dehydrogenase (G6PDH) were prepared from tumour pieces (>100 mg weight, previously excised and stored in liquid $N_2$) according to the method of Yasin and Bergel (1965). Three- to four-$\mu$l samples were applied to analytical Cellogel strips (5·7 × 14 cm, Whatman Lab. Sales, Maidstone, Kent). Both human LDH and G6PDH isoenzymes were separated by flat-bed electrophoresis at 200 V for 95 min at 2°C. LDH isoenzymes were visualized by incubation in a solution consisting of 1 ml 1M lithium lactate, 10 mg nicotinamide adenine dinucleotide (NAD), 3 mg MTT(3-(4,5-dimethyl thiazolyl-2)-2,5-diphenyl tetrazolium bromide), 4·3 ml water and 0·5 mg phenazine methosulphate (PMS) at 37°C for 5–10 min. G6PDH isoenzymes were stained in a solution containing 30 ml 0·1M Tris-HCl buffer (pH 8·0) 10 mg NADP, 6 mg MTT, 30 mg glucose-6-phosphate and 0·5 mg PMS at 37°C for up to 30 min. (All chemicals were obtained from Sigma London Chemical Co.)

*Chromosome analysis.*—Single-cell suspensions from passaged tumour material were prepared by trypsinization. These were incubated with 0·4 $\mu$g/ml Colcemid (CIBA) for 3h and 18h periods. Cultures were harvested, slides prepared and chromosomes banded using a modified method of Reeves (1973).

*Tumour lines.*—The 6 human colorectal tumour lines used in this study were established from patients who had previously received no chemotherapy or radiotherapy. Details of the patients studied, the location and histological classification of the tumours, and the number of passages studied in both male and female mice for each tumour line, are shown in Table I. For ease of reference, the common first two letters in the symbols for each tumour (HX) will be omitted from the rest of this paper.

### RESULTS

Growth was obtained from 6 out of 9 transplanted human colorectal tumours, and between 4 and 10 serial passages in both male and female immune-deprived mice were studied (Table I). Tumour lines $AC_4$ and $HC_1$ were maintained only in male and female mice respectively.

### *Retention of histological integrity*

The human tumours and their corresponding xenografts ranged from moderately well to poorly differentiated. Fibrotic material observed in sections from the human primary tumours at surgical resection was not observed in xenograft tumours. Passaged tumours contained large central necrotic areas, although they were highly cellular toward the tumour periphery.

*Tumour line BR.*—The glands within the moderately well-differentiated human primary tumour were in some areas lined by single layers of epithelial cells showing irregularly placed nuclei. In other areas there were several layers of cells (stratified) interspersed with smaller glands. Some regions showed better differentiation than others. On Passage 1 in both male and female mice, histological integrity tended to reflect the more poorly differentiated areas of the human primary tumour. The formation of cysts was evident, showing thin bands of cells containing the glands surrounding much larger areas of necrosis. In some areas the cellular bands were thicker than in others, probably due to the outward proliferation of the thinner-walled cysts. This type of structure then appeared to remain for the 5 passages studied in both host sexes.

TABLE I.—*Clinical Data on Each Patient and Tumour, and Number of Passages in both Male and Female Immune-deprived Mice.*

| Tumour | Patient's age and sex | | Operation and date | Tumour location Histological type | Postoperative course | No. of passages in mice | |
|---|---|---|---|---|---|---|---|
| | | | | | | ♂ | ♀ |
| $HXSR_1$* | 71 | ♀ | 15.11.74. Abdomino-perineal resection of rectum | Rectum Mod. well. diff. | Lost to follow-up | — | — |
| HXBR | 51 | ♀ | 26.11.74. Abdomino-perineal resection of rectum | Rectum Mod. well. diff. | 30.9.75. Alive and well. No chemo-therapy. No radiotherapy | 5 | 5 |
| HXFC* | 78 | ♂ | 20.11.74. Abdomino-perineal resection | Sigmoid colon Mod. well. diff. | 13.3.75. Alive and well. No chemo-therapy. No radiotherapy | — | — |
| $HXAC_4$ | 80 | ♀ | 24.1.75. Right hemicolectomy | Caecum Mod. well. diff | Lost to follow-up | 4 | — |
| $HXHC_1$ | 68 | ♀ | 4.12.74. Removal of caecum. End-to-end union between small and large bowel | Ascending colon Mod. well. diff. | 22.1.76. Alive and well. No chemo-therapy. No radiotherapy | — | 10 |
| $HXGC_3$ | 61 | ♂ | 15.1.75. Transverse colectomy with end-to-end anastomosis | Transverse colon Poorly diff. | 7.5.76. Alive and well. No abdominal masses felt | 10 | 10 |
| $HXVRC_5$ | 72 | ♂ | 28.2.75. Right hemicolectomy | Caecum Poorly diff. | Lost to follow-up | 8 | 10 |
| $HXPR_2$* | 68 | ♀ | 13.5.74. Laparotomy, by-pass colostomy 15.11.74. Removal of rectal tumour | Rectum Poorly diff. | Patient died | — | — |
| $HXELC_2$ | 83 | ♀ | 20.12.74. Right hemicolectomy | Caecum Poorly diff. | 3.4.75. Supraclavicu-lar node involve-ment. 5-FU treatment. Disease progressing | 10 | 10 |

* No takes resulted from tumour transplants.

(a)

(b)

FIG. 1. (a)—The human primary tumour from $HC_1$ showing a moderately well-differentiated structure. H. & E. ×306. (b) The histological integrity is maintained in Tumour Line $HC_1$ on Passage 10 in female mice. H. & E. ×306.

*Tumour line $AC_4$.*—The moderately well-differentiated human tumour was less well differentiated than BR. Glands were small, and surrounding cells stratified. Few glands were lined by a single layer of cells. Cyst formation was present on Passage 1 and subsequent passages, and the histological integrity of the human primary appeared to be maintained.

*Tumour line $HC_1$.*—The selected specimen from the human primary tumour was

FIG. 2.—(a) The primary tumour of Line $ELC_2$ showing densely packed "signet ring" cells. H. & E. ×759. (b) High-power magnification of a Passage 10 tumour from Line $ELC_2$ maintained in female mice, showing preservation of the "signet ring" appearance. H. & E. ×759.

extremely fibrotic. The moderately well-differentiated structure is shown in Fig. 1(a). When transplanted, tumours grew in female mice only, and 10 passages were studied. Cyst formation was present, and viable areas reflected similar histological patterns to those seen in the human primary (Fig. 1(b)). Implantation of freeze-thawed pieces of the human primary tumour, previously stored in liquid $N_2$,

TABLE II.—*HID/AB Sequence Demonstrating Sulphated Sialic Acid Mucins in Human Primary and Passaged Tumour Specimens*

| Tumour code | sex | Human tumour | Passage 1 | 2 | 3 | 4 | 5 | 6 | 7 | 8 | 9 | 10 |
|---|---|---|---|---|---|---|---|---|---|---|---|---|
| BR | ♂<br>♀ | +<br>− | +++<br>− | +++<br>−− | ++<br>− | +++<br>−− | +++<br>− | | | | | |
| | ♀ | | +++<br>−− | +++<br>− | +++<br>− | +++<br>− | +++<br>− | | | | | |
| $AC_4$ | ♂<br>♀ | + | ++ | ++ | ++ | ++ | | | | | | |
| $HC_1$ | ♂ | + | | | | | | | | | | |
| | ♀ | | ++ | ++ | ++<br>− | ++<br>− | ++<br>− | ++<br>− | ++ | ++ | ++ | ++ |
| $GC_3$ | ♂ | −−− | −−− | +++<br>−−− | ++<br>−−− | ++<br>−−− | ++<br>−−− | +++<br>−−− | +++<br>−−− | +<br>−−− | +<br>−− | ++<br>−−− |
| | ♀ | | −−− | +<br>−−− | ++<br>−−− | ++<br>−−− | +++<br>−− | ++<br>−−− | ++<br>−− | ++<br>−− | ++<br>−− | +++<br>−−− |
| $VRC_5$ | ♂ | − | −− | −− | −− | −− | −− | −− | −− | −− | | |
| | ♀ | | −− | − | −− | −− | − | − | − | −− | −− | −− |
| $ELC_2$ | ♂ | ++<br>−−− | ++<br>−−− | +++<br>−−−− | +++<br>−−−− | +++<br>−−−− | ++<br>−−−− | +++<br>−−−− | ++++<br>−−−− | ++++<br>−−−− | ++++<br>−−−− | ++++<br>−− |
| | ♀ | | ++<br>−−− | +++<br>−−−− | +++<br>−−−− | +++<br>−−−− | +++<br>−−− | +++<br>−−− | +++<br>−− | +++<br>−− | +++<br>−− | ++<br>−− |

| | Sulphomucins (black/brown) | Sialo mucins (blue) |
|---|---|---|
| Trace amounts | + | − |
| Small amounts | ++ | −− |
| Moderate amounts | +++ | −−− |
| Copious amounts | ++++ | −−−− |

TABLE III.—*AB pH 2·5/PAS Sequence Demonstrating Neutral and Acid Mucins in Human Primary and Passaged Tumour Specimens*

| Tumour code | sex | Human Tumour | Passage 1 | 2 | 3 | 4 | 5 | 6 | 7 | 8 | 9 | 10 |
|---|---|---|---|---|---|---|---|---|---|---|---|---|
| BR | ♂ | +<br>− − | +<br>− − − | +<br>− − − | +<br>− − − | +<br>− − − | +<br>− − − | | | | | |
| | ♀ | | +<br>− − − | +<br>− − − | +<br>− − − | +<br>− − − | +<br>− − − | | | | | |
| $AC_4$ | ♂ | + +<br>− | + +<br>− | + +<br>− − | + +<br>− − | + +<br>− − | | | | | | |
| | ♀ | | | | | | | | | | | |
| $HC_1$ | ♂ | +<br>− | | | | | | | | | | |
| | ♀ | | +<br>− − | +<br>− − | +<br>− − | +<br>− − | +<br>− − | +<br>− − | +<br>− − | +<br>− − | +<br>− − | +<br>− − |
| $GC_3$ | ♂ | + +<br>− − − | + +<br>− − − | + +<br>− − − | + +<br>− − − | + +<br>− − − | + +<br>− − − | +<br>− − − | +<br>− − − | + +<br>− − − | + +<br>− − − | +<br>− − − |
| | ♀ | | + +<br>− − − | + + +<br>− − − | + + +<br>− − − | + + +<br>− − − | + + +<br>− − − | + + +<br>− − − | + + +<br>− − | + + +<br>− − | + + +<br>− − − | + + +<br>− − − |
| $VRC_5$ | ♂ | +<br>− | +<br>− − | + +<br>− − | + +<br>− − | + +<br>− − | + +<br>− − | + +<br>− − | + +<br>− − | + +<br>− − | | |
| | ♀ | | +<br>− | +<br>− − | + +<br>− − | + +<br>− − | + +<br>− − | + +<br>− − | +<br>− − | +<br>− − | + +<br>− − | + +<br>− − |
| $ELC_2$ | ♂ | + +<br>− − − | + +<br>− − − | + +<br>− − − − | + + +<br>− − − − | + +<br>− − − − | + + +<br>− − − − | + + +<br>− − − − | + +<br>− − − − | + +<br>− − − − | + + +<br>− − − − | +<br>− − − − |
| | ♀ | | + + +<br>− − − − | + + +<br>− − − − | + + +<br>− − − − | + + +<br>− − − − | + + +<br>− − − − | + + +<br>− − − − | + +<br>− − − − | + +<br>− − − − | + + +<br>− − − − | + +<br>− − − − |

| | Neutral (magenta) | Acid (blue) |
|---|---|---|
| Trace amounts | + | − |
| Small amounts | + + | − − |
| Moderate amounts | + + + | − − − |
| Copious amounts | + + + + | − − − − |

into male hosts resulted in the growth of a single tumour with histological characteristics differing from those observed during the growth of the tumour line in female hosts.

*Tumour line $GC_3$*.—The human primary was highly cellular and poorly differentiated. Glands were variable in size, and surrounding cells were densely packed. Glandular density appeared to vary in different tumour areas. This structure was maintained throughout 10 serial passage in both host sexes.

*Tumour line $VRC_5$*.—The poorly differentiated human primary showed a highly cellular pattern. Glands were small and were of more frequent occurrence in some areas than in others. Eight passages in male mice and 10 in female hosts were studied. Histological integrity was preserved.

*Tumour line $ELC_2$*.—This, the least differentiated tumour of the series, showed no glandular differentiation. The primary comprised many densely packed "signet ring" cells showing large accumulations of mucin in the cytoplasm, with the compression of the nuclei on to their cell walls (Fig. 2(a)). The selected specimen was highly cellular, and in some areas cells were seen to be arranged in clusters, surrounded by mucin lakes. For up to 10 serial passages in both male and female mice the same histological patterns were evident (Fig. 2(b)).

*Retention of epithelial mucin secretion*

A subjective assessment of the quantities of sialic acid, sulphated and neutral mucins produced by each human primary tumour and the corresponding xenografts during serial passage was made. Results are presented in Tables II and III. Table II demonstrates the HID/AB sequence applied for the differential demonstration of sialic acid and sulphated mucins. Sulphomucins were scored using the symbols + to ++++, showing a range of production from traces to copious amounts. Sialic acid mucins were scored using the symbols – to – – – –. A similar assessment of acid (consisting of both sialic acid and sulphated mucins) *vs* neutral mucin production using the AB pH 2·5/PAS sequence was made, again using – and + respectively as symbols of grading (Table III).

A comparison of the human primary tumours with human normal colonic mucosa showed that mucin secretion was reduced in all tumours except $ELC_2$. In passaged tumours of each tumour line mucin secretion was elevated, probably due to the increased cellularity of xenograft tumours compared to their human primaries. In Tumour Lines BR, $AC_4$ and $HC_1$ the secretion comprised mainly sulphated mucin admixed with some neutral mucosubstance during serial passage. The poorly differentiated Tumour Lines $GC_3$ and $ELC_2$ produced all 3 mucins, whereas $VRC_5$ demonstrated only sialic acid and neutral products. Tumour Lines BR, $AC_4$, $HC_1$ and $VRC_5$ (but not $GC_3$ or $ELC_2$) all demonstrated a decrease in intracellular staining intensity using the AB pH 2·5/PAS sequence, after pretreatment with salivary diastase, showing the presence of glycogen deposits. Extracellular secretions of mucin located within the acini were unaffected by this procedure. Tumour Lines $GC_3$ and $ELC_2$, in particular, showed variations in the pattern of mucin secretion from passage to passage in both host sexes, and often within different areas of the same tumour. In Tumour Line $GC_3$, sulphomucin not demonstrable within the selected human primary tumour specimen of Passage 1 tumours was present during subsequent passages.

*Production of carcinoembryonic antigen (CEA)*

Table IV demonstrates the quantity of CEA produced by the human primary tumours, and by xenografts on selected passages in both male and female immune-deprived mice, expressed as ng CEA/mg tumour tissue. The levels of CEA observed are dependent upon the numbers of viable cells within the chosen specimens. Human

TABLE IV.—*The Production of CEA (ng/mg tumour tissue) in Human Primary and Xenograft Tumours*

| Tumour line | Sex | BR | | $AC_4$ | | $HC_1$ | | $GC_3$ | | $VRC_5$ | | $ELC_2$ | |
|---|---|---|---|---|---|---|---|---|---|---|---|---|---|
| | | Pass. | CEA | Pass. | CEA | Pass. | CEA | Pass. | CEA | Pass. | CEA | Pass. | CEA |
| Human tumour | — | — | 5 | — | — | — | 23 | — | 0·1 | — | 2 | — | >8 |
| Early passage | ♂ | 1 | 157 | 1 | >39 | — | — | 1 | 44 | 1 | 137 | 1 | 101 |
| Later passage | | 4 | 764 | 3 | >23 | — | — | 7 | 39 | 7 | 102 | 9 | 49 |
| Early passage | ♀ | — | — | — | — | 2 | 146 | 1 | 11 | 1 | 52 | 1 | 20 |
| Later passage | | 3 | 201 | — | — | 8 | 177 | 8 | 42 | 8 | 22 | 8 | 119 |

FIG. 3.—Electrophoretic separation of LDH isoenzymes from CBA/lac mouse skin and tumour-bearing human colon, demonstrating species-specific patterns. Both human and murine variants may be identified from a mixture.

primary tumours contained fibrotic material, and passaged tumours, large centrally necrosed areas. Consequently the data act purely as an indication of the production or non-production of CEA. Xenograft tumour material for the assay was taken from peripheral and therefore cellular areas. Assayed levels in these tumours therefore should be higher than those obtained for the human primary specimens. Insufficient material was available for the assay of human primaries $AC_4$ and $ELC_2$. However, CEA was produced by all selected passaged tumours from each tumour line, from both male and female mice. Results for the human primary tumours are consistent with those of Khoo *et al.* (1973) who found the concentration of CEA in adenocarcinoma of the colon ranged from 0·23 to 2·73 ng/mg tumour tissue. They also demonstrated the presence of CEA in normal human colon, although in reduced amounts (0·028–0·086 ng/mg tissue).

*Retention of human isoenzymes.*—LDH and G6PDH have been found to exist in species-specific forms. Human tissues such as heart and skeletal muscle demonstrate 5 LDH variants (Leese, 1969) whereas up to 15 variants have been demonstrated in mice (Shaw and Barto, 1963), although only 5 have been found in mouse epidermis (Quevedo *et al.*, 1975).

Fig. 3 shows the electrophoretic separation of the 5 LDH variants from CBA/lac mouse skin, tumour-bearing human colon, and a mixture of solutions prepared from the two tissues. Human variants are designated according to the M/H nomenclature (Kaplan, 1963) where the pure $H_4$ variant is the one moving most anodically, and pure $M_4$ most cathodically, during

FIG. 4.—LDH isoenzymes in Tumour Line $ELC_2$, compared with those in mouse skin and human tumour.

electrophoresis. The variants $MH_3$, $M_2H_2$ and $M_3H$ are located between the two pure tetrameric forms of LDH. Murine isoenzymes have been named LDH-1 to 5, where LDH-1 is located nearest to the anode during electrophoresis. The mobilities of murine LDH-1 and LDH-4 are similar to those of human $H_4$ and $M_2H_2$ variants, although murine LDH-2, LDH-3 and LDH-5 may all be identified clearly in a mixture.

All the passaged tumours of each tumour line maintained human LDH isoenzyme patterns. Tumour Line $ELC_2$ is shown in Fig. 4. Although murine LDH-5 was consistently present, the human variants predominated. The single tumour which arose in Passage 1 of Tumour Line $HC_1$ in male hosts from the implantation of freeze-thawed tumour material and whose histology was found to differ from that of the tumours maintained in female hosts showed mouse-specific LDH only, from analyses made on the Passage 2 generation.

The electrophoretic mobility patterns for G6PDH are also species-specific. Human G6PDH has been found to have a slower mobility than that observed for the rat (Beutler and Collins, 1965) the hamster (Goldenberg, Bhan and Pavia, 1971) or the mouse (Povlsen *et al.*, 1973).

In each tumour line, two G6PDH variants were present in the passaged tumour material, consistent with the presence of both human and murine cells. In Tumour Line $HC_1$ the single tumour arising in male hosts and showing mouse-specific LDH on Passage 2 also demonstrated mouse-specific G6PDH from analyses on the Passage 2 generation.

*Retention of human chromosomes*

Tumours from both male and female hosts were analysed. They were taken from Tumour Lines $HC_1$, $GC_3$, $VRC_5$ and $ELC_2$

between Passages 7 and 11, and BR and $AC_4$ in Passages 3 and 4. Detailed karyotyping will be described elsewhere (Reeves and Houghton, 1977) although preparations from all passaged tumour material showed characteristically human metaphase spreads. Occasionally, murine metaphase spreads were also observed, although these were infrequent. Tumour Lines $AC_4$, $GC_3$, $VRC_5$ and $ELC_2$ produced some obvious marker chromosomes, a feature characteristic of human colorectal tumours (Xavier *et al.*, 1974). Two marker chromosomes present in later passages of $ELC_2$ were also present on Passage 2 from female hosts. The tumours of line $HC_1$ on Passages 1 and 2 in male hosts produced characteristic murine metaphase spreads.

### DISCUSSION

The histological integrity observed within each human primary tumour was maintained during serial passage in both male and female immune-deprived mice. There appeared to be no preferential treatment by either host sex in the preservation of these features. Cobb (1973) over the first two passages in immune-deprived mice, and Povlsen and Rygaard (1971) in up to 9 transplant generations in nude mice, also found that the histological characteristics of human colorectal tumours were retained. In this tumour series, Line BR, although moderately well differentiated, histologically resembled the more poorly differentiated areas of the human primary tumour. Similar observations were made by Cobb (1973) in one tumour line, and Povlsen and Rygaard (1971) found that a poorly differentiated human colonic tumour assumed an anaplastic appearance upon implantation into nude mice. It is possible that this apparent selection was caused by the implantation of non-representative areas of the human primary tumour. However, as (as in this study) tumour material was implanted bilaterally into 40 mice on Passage 1, this would therefore appear unlikely. Whether or not all types of cellular arrangements may be maintained in immune-deficient mice remains a question to be answered. Certainly, tumours proving to be non-transplantable have not always been well differentiated types in this and in other studies (Povlsen and Rygaard, 1971). A poorly differentiated human rectal carcinoma in this series ($PR_2$, Table I) in addition to two tumours with moderate differentiation, failed to grow in mice.

Xenograft tumours of each tumour line, and from both male and female hosts, also demonstrated epithelial mucins of the type secreted by their corresponding human primary tumours. In colorectal carcinoma, all 3 epithelial mucin types have been demonstrated (Gad, 1969; Filipe, 1969; Subbuswamy, 1971; Cooper, 1974) although there appears to be no set pattern of mucin production.

The quantity and type of mucin produced in the current xenograft tumour series was seen to vary between different passages of the same tumour line, and even within different areas of the same tumour. This was particularly evident in $GC_3$ and $ELC_2$ tumours. It has previously been shown that the mucin type changes qualitatively between the lower and upper crypts and the surface epithelium of normal human colonic mucosa (Filipe, 1969). It is possible, therefore, that each mucin-producing cell has the capacity to produce either one or a mixture of these mucins, where the particular mucin produced is under specific genetic regulation. In colorectal carcinoma, both mucous and non-mucous areas have been found to occur within the same tumour (Cooper, 1974). In carcinoma of the stomach, variations in sulphated and non-sulphated acid mucin content have been identified in different areas of the same tumour (Goldman and Ming, 1968). It is possible that such features reflect an instability in genetic expression in tumours and that the variations observed in $GC_3$ and $ELC_2$ human tumour xenografts would be a feature normally observed in the clinical situation.

It has been shown that immunedeprived mice are able to maintain the growth of a wide range of histological tumour types, which retain the mucin- and CEA- producing characteristics of their human primary tumours. In addition, retention of human enzyme systems and a human chromosome constitution is particularly important, especially during the use of antimetabolites, where blockade of a single enzyme or activation of the agent in a specific metabolic pathway may be important. The two enzymes assayed, namely LDH and G6PDH, displayed human isoenzyme patterns on each passage of each tumour line, thus yielding strong support for the maintenance of a human metabolism in this system. The presence of human chromosomes demonstrated the continued existence of human cells even after serial passage. The presence of murine tissue has been identified from mouse-specific LDH and G6PDH, and an occasional murine metaphase spread from chromosome analyses. Although the contribution of mouse tissues to the xenografts has not been examined in this study, it is probable that connective tissues and adhering blood vessels may be involved.

The induction of a murine tumour was identified in this system in one instance after the implantation of human primary tumour material. Hence in the use of xenograft systems it is necessary to establish routinely the continued presence of human characteristics during the serial passage of each tumour line, using techniques such as isoenzyme and/or chromosome analyses. This is particularly relevant where anaplastic or very poorly differentiated human tumours are involved. In such cases, passaged tumours may continue to resemble morphologically the human primary tumour, even after the formation of a hybrid (Goldenberg *et al.*, 1971).

We wish to thank Dr A. Mackay, Dr R. L. Carter, Dr C. L. Leese and Dr B. R. Reeves for helpful discussion and advice during the current investigations, and to extend our thanks to Miss S. Clinton for her technical assistance, and Miss S. Carter for the assay of CEA.

### REFERENCES

BEUTLER, E. & COLLINS, Z. (1965) Hybridization of Glucose-6-phosphate Dehydrogenase from Rat and Human Erythrocytes. *Science, N.Y.*, **150**, 1306.

COBB, L. M. (1973) The Behaviour of Carcinoma of the Large Bowel in Man Following Transplantation into Immune-deprived Mice. *Br. J. Cancer*, **28**, 400.

COBB, L. M. & MITCHLEY, B. C. V. (1974) Development of a Method for Assessing the Antitumor Activity of Chemotherapeutic Agents using Human Tumor Xenografts. *Cancer Chem. Repts.*, **58**, 645.

COOPER, D. J. (1974) Mucin Histochemistry of Mucous Carcinomas of Breast and Colon and Non-neoplastic Breast Epithelium. *J. clin. Path.*, **27**, 311.

FILIPE, M. I. (1969) Value of Histochemical Reactions for Mucosubstances in the Diagnosis of Certain Pathological conditions of the Colon and Rectum. *Gut*, **10**, 577.

GAD, A. (1969) A Histochemical Study of Human Alimentary Tract Mucosubstances in Health and Disease. I. Normal and Tumours. *Br. J. Cancer*, **23**, 52.

GOLDENBERG, D. M., BHAN, R. D. & PAVIA, R. A. (1971) *In vivo* Human–Hamster Somatic Cell Fusion Indicated by Glucose-6-phosphate Dehydrogenase and Lactate Dehydrogenase Profiles. *Cancer Res.*, **31**, 1148.

GOLDMAN, H. & MING, S. C. (1968) Mucins in Normal and Neoplastic Gastrointestinal Epithelium. *Archs Path.*, **85**, 580.

HOUGHTON, P. J., HOUGHTON, J. A. & TAYLOR, D. M. (1977) Effects of Cytotoxic Agents on TdR Incorporation and Growth Delay in Human Colonic Tumour Xenografts. *Br. J. Cancer*, **36**, 206.

HOUGHTON, J. A. & TAYLOR, D. M. (1976) Effects of Serial Passage on Human Tumour Xenografts Grown in Immune-deprived Mice. *Br. J. Cancer*, **34**, 313.

KAPLAN, N. O. (1963) Symposium on Multiple Forms of Enzymes and Control Mechanisms. *Bacteriol. Rev.*, **27**, 155.

KHOO, S. K., WARNER, N. L., LIE, J. T. & MACKAY, I. R. (1973) Carcinoembryonic Antigen Activity of Tissue Extracts: a Quantitative Study of Malignant and Benign Neoplasms, Cirrhotic Liver, Normal Adult and Fetal Organs. *Int. J. Cancer*, **11**, 681.

KOPPER, L. & STEEL, G. G. (1975) The Therapeutic Response of Three Human Tumor Lines Maintained in Immune-suppressed Mice. *Cancer Res.*, **35**, 2704.

LAURENCE, D. J. R., STEVENS, U., BETTELHEIM, R., DARCY, D., LEESE, C., TURBERVILLE, C., ALEXANDER, P., JOHNS, E. W. & NEVILLE, A. M. (1972) Role of Plasma Carcinoembryonic Antigen in Diagnosis of Gastrointestinal, Mammary and Bronchial Carcinoma. *Br. med. J.*, iii, 605.

LEESE, C. L. (1969) Enzymes and Isoenzymes. In *Homeostatic Regulators*. Ed. Wolstenholme, G. E. W. and Knight, J. London: Churchill. p. 144.

PICKARD, R. G., COBB, L. M. & STEEL, G. G. (1975) The Growth Kinetics of Xenografts of Human Colorectal Tumours in Immune-deprived Mice. *Br. J. Cancer*, **31**, 36.

POVLSEN, C. O., FIALKOW, P. J., KLEIN, E., KLEIN, G., RYGAARD, J. & WEINER, F. (1973) Growth and Antigenic Properties of a Biopsy-derived Burkitt's Lymphoma in Thymus-less (Nude) Mice. *Int. J. Cancer*, **11**, 30.

POVLSEN, C. O. & RYGAARD, J. (1971) Heterotransplantation of Human Adenocarcinomas of the Colon and Rectum to the Mouse Mutant Nude. A Study of Nine Consecutive Transplantations. *Acta. path. microbiol. scand.*, (A), **79**, 159.

QUEVEDO, W. C., BIENIEKI, T. C., HOLSTEIN, T. J. & DYER, H. J. (1975) Lactate Dehydrogenase Isozymes of Mouse Epidermis. *Experientia*, **31**, 1034.

REEVES, B. R. (1973) Cytogenetics of Malignant Lymphomas. Studies Utilizing a Giemsa-banding Technique. *Humangenetik*, **20**, 231.

REEVES, B. R. & HOUGHTON, J. A. (1977) Serial Cytogenetic Studies of Human Colonic Tumour Xenografts. *Br. J. Cancer*, **37** (in press).

SHAW, C. R. & BARTO, E. (1963) Genetic Evidence for the Subunit Structure of Lactate Dehydrogenase Isozymes. *Proc. natn. Acad. Sci. U.S.A.*, **50**, 211.

SPICER, S. S. (1965) Diamine Methods for Differentiating Mucosubstances Histochemically. *J. Histochem. Cytochem.*, **13**, 211.

SUBBUSWAMY, S. G. (1971) Mucosubstances in Neoplasms of the Human Colon and Rectum. *Gut*, **12**, 200.

XAVIER, R. G., PROLLA, J. C., BEMVENUTI, G. A. & KIRSNER, J. B. (1974) Tissue Cytogenetic Studies in Chronic Ulcerative Colitis and Carcinoma of the Colon. *Cancer, N.Y.*, **34**, 684.

YASIN, R. & BERGEL, F. (1965) Lactate Dehydrogenase Isoenzyme Patterns in Human Normal and Malignant Gastric Mucosa. *Eur. J. Cancer*, **1**, 203.