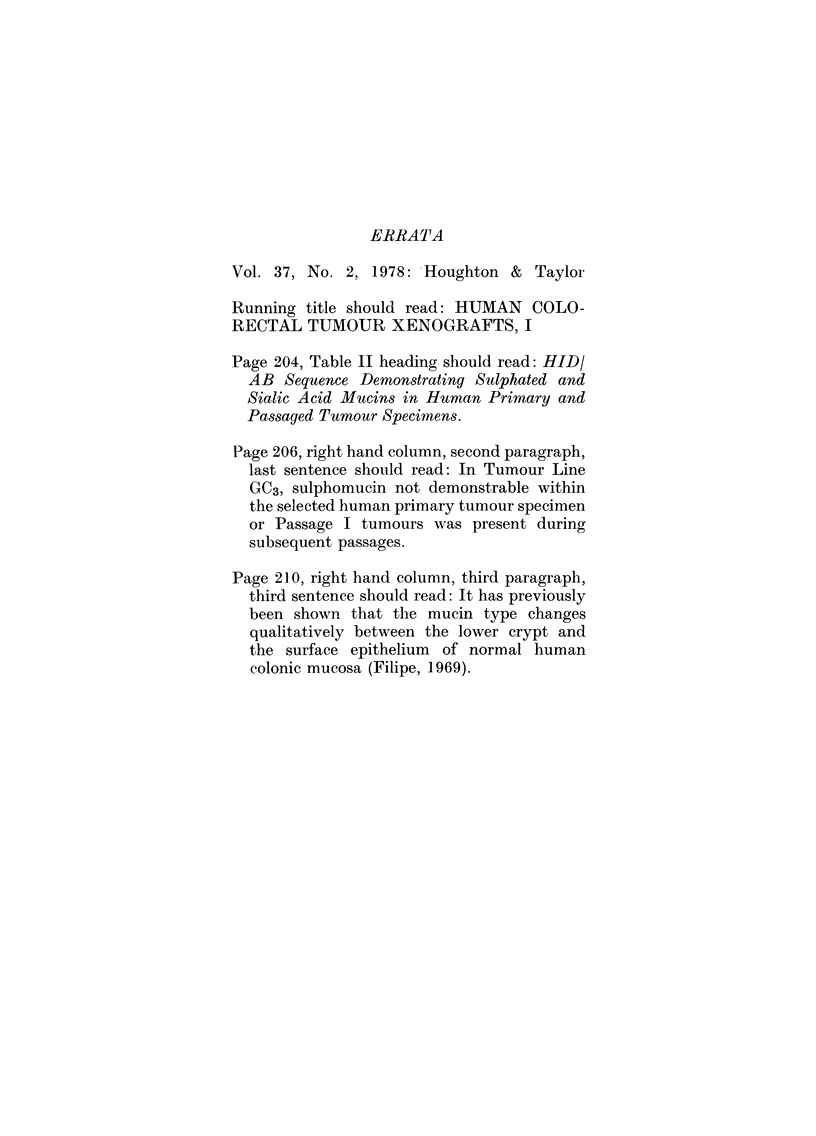# Errata

**Published:** 1978-02

**Authors:** 


### *ERRATA*

Vol. 37, No. 2, 1978: Houghton & Taylor

Running title should read: HUMAN COLORECTAL TUMOUR XENOGRAFTS, I

Page 204, Table II heading should read: *HID/AB Sequence Demonstrating Sulphated and Sialic Acid Mucins in Human Primary and Passaged Tumour Specimens.*

Page 206, right hand column, second paragraph, last sentence should read: In Tumour Line $GC_3$, sulphomucin not demonstrable within the selected human primary tumour specimen or Passage I tumours was present during subsequent passages.

Page 210, right hand column, third paragraph, third sentence should read: It has previously been shown that the mucin type changes qualitatively between the lower crypt and the surface epithelium of normal human colonic mucosa (Filipe, 1969).